# Identification of Potential WSB1 Inhibitors by AlphaFold Modeling, Virtual Screening, and Molecular Dynamics Simulation Studies

**DOI:** 10.1155/2022/4629392

**Published:** 2022-05-13

**Authors:** Ye Weng, Chenghao Pan, Zheyuan Shen, Sikang Chen, Lei Xu, Xiaowu Dong, Jing Chen

**Affiliations:** ^1^The Fourth School of Clinical Medicine, Zhejiang Chinese Medical University, Hangzhou 310053, China; ^2^School of Pharmaceutical Sciences, Zhejiang Chinese Medical University, Hangzhou 310053, China; ^3^Institute of Drug Discovery and Design, College of Pharmaceutical Sciences, Zhejiang University, Hangzhou 310018, China; ^4^Institute of Bioinformatics and Medical Engineering, School of Electrical and Information Engineering, Jiangsu University of Technology, Changzhou 213001, Jiangsu, China

## Abstract

WD40 repeat and SOCS box containing 1 (WSB1) consists of seven WD40 repeat structural domains at the *N*-terminal end and one SOCS box structural domain at the *C*-terminal end. WSB1 promotes cancer progression by affecting the Von Hippel–Lindau tumor suppressor protein (pVHL) and upregulating hypoxia inducible factor-1*α* (HIF-1*α*) target gene expression. However, the crystal structure of WSB1 has not been reported, which is not beneficial to the research on WSB1 inhibitors. Therefore, we focused on specific small molecule inhibitors of WSB1. This study applied virtual screening and molecular dynamics simulations; finally, 20 compounds were obtained. Among them, compound G490-0341 showed the best stable structure and was a promising composite for further development of WSB1 inhibitors.

## 1. Introduction

Metastasis is one of the causes of cancer death [1–3]. However, due to the lack of effective interventions, the study of new antimetastatic targets has become a popular research topic in oncology-related research areas. Tumor-derived blood vessels are unevenly distributed, and their function is abnormally compared to normal blood vessels, leading to the persistence of a hypoxic microenvironment in the tumor, which shows that tumor metastasis is closely related to hypoxia [4, 5]. HIF-l has been reported to regulate various related signaling pathways during the adaptation of tumor cells to the hypoxic environment and plays a crucial role in tumor cell proliferation, angiogenesis, malignant invasion, and metastasis [6–9]. For example, high expression of HIF-l has been associated with local infiltration and distal metastasis of tumors, including colon, prostate, and lung cancers [10, 11].

HIF-1 regulates WSB1 expression. WSB1 increases under hypoxic conditions, and it has been identified to be dependent on HIF-1 [12, 13]. WSB1 has been shown to regulate pVHL protein stability not only under hypoxia but also under normoxia. WSB1 can upregulate HIF-1 through ubiquitination of pVHL [14–16]. Thus, HIF-1 and WSB1 form a positive feedback loop, which provides a strong activation of HIF-1. Under hypoxic conditions, the HIF-l protein transcriptionally activates the E3 ubiquitin ligase family protein WSB1 and induces an increase in its protein expression.

There are many research studies about WSB1 regulating tumor progression [17–21]. WSB1 was found to drive the metastatic potential in osteosarcoma cells which correlated with the pulmonary metastatic potential. WSB1 plays a role in neuroblastoma cell growth and involved in pancreatic cancer progression. Besides, it has also been reported to participate in the carcinogenesis of lung cancer.

Currently, studies on the crystal structure of WSB1 and the inhibition of WSB1 activity by gene silencing and gene mutation techniques are still in progress. There are still many challenges for tumor control, and it is even more important for us to investigate small molecule inhibitors to support the development of subsequent new drugs.

However, in cancer drug discovery, the experimental tests used to examine small molecules are often expensive and laborious [22]. In recent years, artificial intelligence (AI) has provided new opportunities for drug discovery [23–25]. In this study, we applied AlphaFold2 to predict the protein structure of WSB1. After that, molecular dynamics simulations were applied to optimize the structure as well as software to validate the accuracy of the modeled structure, followed by peptide-protein docking and structure-based virtual screening including AutoDock-GPU and Glide. Virtual screening (VS) is a powerful drug discovery tool that takes advantage of high-performance computers to filter compounds by using ligand or structure-based methods [26–28]. Finally, four compounds with different compound scaffolds were selected, namely, G490-0341, G610-0188, Y043-6168, and Y044-5019 compounds. Moreover, the binding mode of compound G490-0341 was investigated, which provided important information for further structural modifications. These results provide relevant information for the study of WSB1 inhibitor drugs and may assist in future drug design.

## 2. Materials and Methods

### 2.1. Protein Structure Acquisition and Evaluation of Repair

We obtained the AlphaFold model from their web page (https://deepmind.com/research) as an initial machine learning-based model. Our latest improved protocol based on molecular dynamics simulations was applied to the protein model. This approach is an improved version of the protocol we used previously during CASP13. The repaired protein structure was further processed by the Protein Preparation Wizard for modules including hydrogenation, redistribution of bond levels, readdition of side-chain residues, recovery of selenomethionine, and specifying conditions such as the protonated state pH of the protein. Finally, molecular dynamics simulations of 200 ns were performed to eliminate the irrational conformation in the structure. To fix the irrational factors in the confirmation, we performed longer (200 ns) simulations at 300 K.

### 2.2. Protein Peptide Docking

Molecular docking was performed in Maestro using GlideSP. First, the protein was prepared for docking using an MOE's quick prep tool, which included correcting structural issues, protonating the structure, removing unbound water molecules, and minimizing the structure to a specified gradient to make the pocket available for docking of the new molecule. The original ligand (D2) was used to define the binding site of the WSB1 active pocket. The LigPrep module was used to prepare the WSB1 inhibitor. After preparation, the ligand-docking module was used to perform the docking work. In the docking parameters, the maximum output confirmation number was set to 20 to improve the accuracy of docking. The molecular conformation with the highest docking score was selected for analysis. The results can be displayed in a ligand interaction diagram.

### 2.3. Virtual Screening

The ChemDiv database is a commercial small molecule database from ChemDiv Inc. (ChemDiv) containing over 1.6 million compounds as screening libraries.

In recent years, there has been an increasing interest in applying various methods to improve its accuracy in molecular docking and eventually to increase the discriminative ability of molecular docking to efficiency. Here, we report a virtual screening campaign for WSB1 inhibitors on the ChemDiv library through a novel selection strategy.

The virtual screening workflow includes AutoDock-GPU, HTVS, SP, and XP models.

First, the first round of screening of ChemDiv was performed by AutoDock-GPU, and the top 10,000 molecules were selected for scoring. After that, the screening was performed by Glide-dock-SP with 5 docking conformation parameters, and 15,000 molecules were obtained for the next round of Glide SP accuracy screening. To improve the screening accuracy, the docking program was reprepared by LigPrep for better adaptation to Glide SP; docking screening was performed by Glide SP for compounds and WDR5 proteins with the highest output confirmation set to 20, and the remaining parameters were kept constant. The final 20 top scoring molecules were obtained, and the combinatorial pose metadynamics analysis was performed.

### 2.4. Binding Pose Metadynamics

The combination of WSB1 and D2 was studied by using three 10 ns independent mild metadynamics simulations of Desmond 39, version 2.3 (Schrödinger, LLC). Metadynamics simulations are a widely used enhanced sampling method for sampling the free energy landscape. Schrödinger's binding pose metadynamics (BPMD) is a variant of metadynamics that samples the motion of ligands in and around their binding modes to use metadynamics methods to determine the relative stability of different binding modes. In this simulation, the biased collective variable is defined as the distance between the center of mass of the ligand molecule and the ligand-binding residues, i.e., Arg174, Arg315, and TYR218, used for WSB1 binding simulations. The initial Gaussian peak height and goodness-of-fit parameters were set to 0.1 and 2.4 kcal/mol, respectively.

In this experiment, we first induced fit docking of the 20 molecules obtained from the virtual screening and then performed BPMD with the 20 poses obtained from the induced fit docking. The stability of the resulting poses was assessed based on the PoseScore. The PoseScore is the root mean square value of the atomic coordinates of the ligand relative to the initial ligand weight. A PoseScore ≤2 Å was considered stable (this RMSD value is often used as a threshold to define the success of prospective docking simulations). The results were analyzed by PersScore, which indicates the strength of the hydrogen bond formed between the ligand and the protein residues. If 60% of the hydrogen bonds were retained during the simulation (e.g., PersScore ≥0.6), this was considered as a sign of good persistence. Finally, compound G490-0341 showed the best stable structure and was obtained as a promising compound for further development of WSB1 inhibitors.

## 3. Results and Discussion

### 3.1. AlphaFold2 Protein Structure Prediction

Jinxin Che et al. found that 5,6-bis (4-methoxy-3-methyl phenyl) pyridine-2-amine acted as a degradation agent to inhibit cancer cell metastasis [18]. However, research on inhibitors targeting the WSB1 axis is still ongoing, so we put more emphasis on drug discovery specifically for WSB1 to identify potential inhibitors. Currently, the crystal structure of WSB1 has not been reported. In this study, we applied *α*-folding deep learning algorithms to predict the structure of WSB1.

AlphaFold2 is artificial intelligence for protein structure prediction, which is a new neural network-based model that can predict protein structures with atomic-level accuracy [29, 30]. In recent years, artificial intelligence and machine learning techniques have played a crucial role in drug discovery and development [31–33]. The neural network of AlphaFold2 can predict the structure of a typical protein in minutes, as well as larger proteins, such as a protein containing 2180 amino acids without a homologous structure. The model provides accurate predictions of the reliability of its predictions on a per amino acid basis. For this experiment, the prediction model was downloaded from the AlphaFold protein structure database [34].

### 3.2. Molecular Dynamics Simulation of WSB1

**Figure 1:**
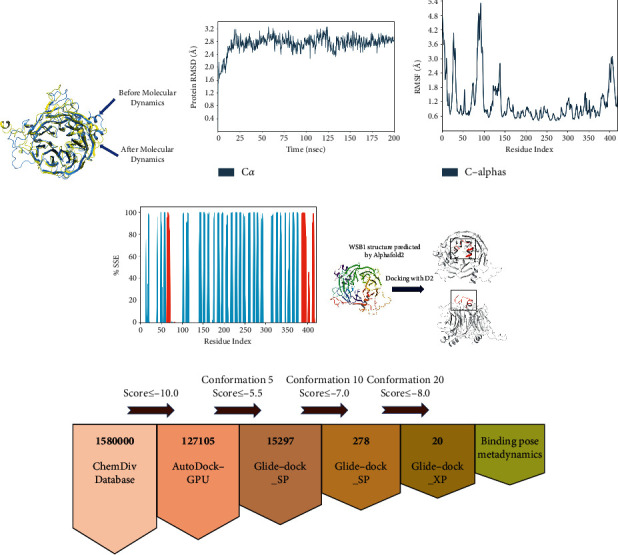
Molecular dynamics simulation results of WSB1. (a) The RMSD plot of a protein. (b) The RMSF plot of a protein 1. The blue line indicates the simulation results of a protein. (c) The SSF plot of a protein. (e) Docking results of the WSB1 protein with D2. The structure of the WSB1 protein is predicted by using AlphaFold2. There are two forms of WSB1 protein docking with D2, and the red part in the black box is D2. (f) The workflow of virtual screening.

We performed molecular dynamics (Figure 1(b)) to enable the repair of the irrational factors in the structure of WSB1 predicted by AlphaFold [35]. After 200 ns MD simulations, we obtained the repaired structure as well as RMSD and RMSF. Figure 1(b) shows the results of the RMSD analysis of the WSB1 interface. WSB1 reached equilibrium after 25 ns simulations and reached a stable conformation below 1 Å, which was acceptable. Meanwhile, the residues of WSB1 were relatively unstable and prone to displacement (Figure 1(c)). The secondary structure of WSB1 (Figure 1(d)) has 6.67% *α*-helix and 34.64% *β*-strand.

### 3.3. WSB1 Docking with Ligands

It was reported that thyroid-hormone-activating type 2-iodothyronine deiodinase (D2) expression was associated with activation of serum thyroid hormone, (de)ubiquitinase ubiquitin-specific peptidase 33, WD repeat sequence, and SOCS box-containing 1 (WSB1), correlated ions of cytokine expression, and inflammatory pathways [36]. To further explore inhibitors targeting WSB1, we hypothesized that D2 docking sites with WSB1 were very promising docking sites. We performed protein-peptide docking of WSB1 and D2 peptides using Maestro and simulated the binding conformation of WSB1 and D2, which is shown in Figure 1(e). WSB1 is also the E3 ubiquitin ligase for D2 [37]. To position D2 relative to the ECSWSB-1 complex, a new loop of 18 amino acids is identified in D2 by comparing a three-dimensional model of D2 with nonubiquitinated D1 and D3 enzymes. It is shown that a better binding position do exist between the 18 amino acids of D2 and WSB1, and we also find further details of the binding between D2 and WSB1. The loops of 18 amino acids are critical for WSB1 to recognize D2 [38, 39]. D2 is integrated into the model by positioning the 18 amino acid loops proximal to the D-A/B-C side of the WSB1 propeller.

In other WD40 propeller E3 ubiquitin ligases, substrate recognition relies on a “supersite” for protein-protein interactions and most commonly the permeation of the second position of each blade, which is often occupied by an arginine residue that interacts with the phosphate group in the substrate. Arginine occupies three sites in WSB1, namely, Arg174, Arg315, and TYR218 [40, 41]. The study confirmed that the WSB-1 WD40 propeller supersite was essential for D2 recognition. The present study restored the details of WSB1 and D2 binding and provided us with more high-definition views of the two bindings.

### 3.4. Virtual Screening

Based on the above analysis, we conducted virtual screening from the ChemDiv database to find the inhibitors targeting WSB1. The ChemDiv database is a commercially available small molecule database containing over 1.58 million compounds that serves as a screening library reference [42]. The virtual screening workflow based on the ChemDiv database is shown in Figure 1(f). Data are shown in Table 1.

Initially, we screened the ChemDiv compound library of 1.58 million molecules by AutoDock-GPU [43] and selected 127,105 molecules with a score of ≤−10.0 for the next round of Glide SP accuracy screening [44]. After screening, we retained 15,297 molecules with scores ≤−5.5 and then performed a conformational restriction screen test (the highest output confirmation was set to 10, and the remaining parameters were kept constant).

**Figure 2:**
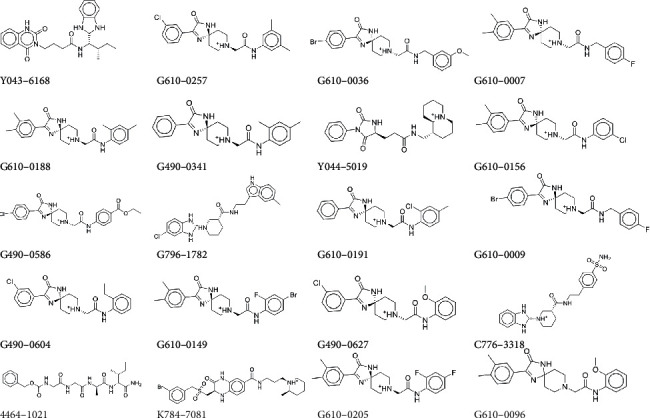
Structures of the 20 compounds obtained by virtual screening.

Finally, we obtained 278 molecules and proceeded to the next screening stage. In the XP mode [45], compounds with Glide SP scores ≤−7.0 were docked to WSB1. This final docking procedure narrowed down our group of compounds to 20 (Glide XP score ≤−8.0), after which we subjected these molecules (Figure 2) to combinatorial pose metadynamics analysis.

### 3.5. Binding Pose Metadynamics

A theoretical strategy able to probe the conformational profile of ligands in the enzyme active site is very important. It is well known that, from a theoretical standpoint, molecular dynamics simulations can be used to evaluate the molecular flexibility of ligands and receptors; however, it is worth mentioning that some conformational changes occur in the time scale of only dozens of nanoseconds, which could compromise the MD simulation viability for virtual screening studies, for instance. In this regard, a theoretical strategy to select promising configurations from the MD simulation is crucial to determine the theoretical accuracy. Hence, great computational effort is necessary to carry out this kind of simulation. Aiming, then, to reduce the number of frames of MD simulations to rationalize the theoretical findings without loss of the relevant information from the simulation, new methods based on the statistical inefficiency such as principal component analysis and wavelet analysis for selecting MD conformations had been developed. Based on the above analysis, we selected binding pose metadynamics [46, 47]. Metadynamics can enhance sampling by allowing efficient back and forth motions across large-free energy barriers, using a more realistic heap of computational resources to sample the relative stability of different binding conformations produced by IDFD while still maintaining full atomic resolution [48, 49]. In contrast, Schrödinger's binding pose metadynamics (BPMD), a variant of metadynamics that samples the motion of ligands in and around their binding modes, aims to use metadynamics methods to determine the relative stability of different binding modes.

Compared to molecular dynamics, BPMD can help select the dominant molecule more accurately and efficiently. In this experiment, three kinetic runs were performed for each of the 20 candidate poses. During the metadynamics calculations of the initial structure, the average RMSD of the 20 candidate poses increased from the beginning to the end of the simulation time and hydrogen bonds were present for finite time of the simulation run. Table 1 summarizes the scores of the 20 candidate poses obtained from the scheme, including PoseScore, PersScore, and ComScore. The stability of the poses is assessed based on the PoseScore, which is the root mean square value of the atomic coordinates of the ligand relative to the initial ligand weight, and a PoseScore ≤2 Å is considered stable. PersScore indicates the strength of the hydrogen bond form between the ligand and the protein residues. If 60% of the total hydrogen bonds are retained during the simulation (e.g., PersScore ≥0.6), it is considered a sign of good persistence.

**Figure 3:**
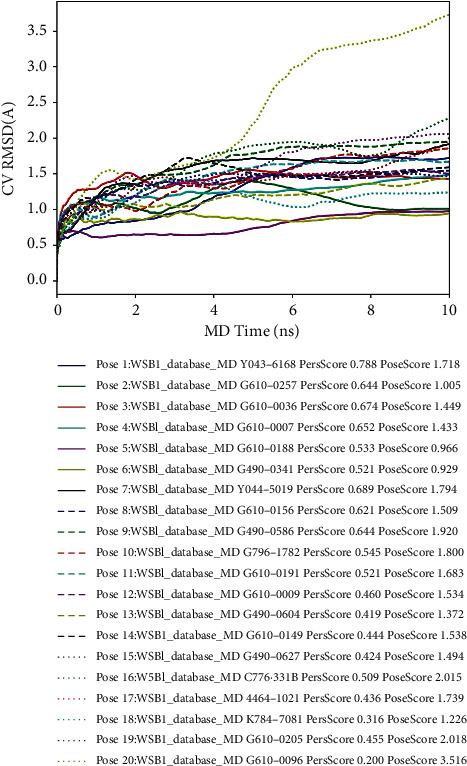
Average RMSD of WSB1 during running 3 × 10 ns metadynamics. The first column of numbers is the number of candidate poses; the second column is the score of PersScore; and the third column is the score of PoseScore.

The docking of the ligand to the WSB1 receptor in pose 6 provided a good example of metadynamics isolating a native-like pose (RMSD of 0.929 Å). This pose was ranked 6th by PersScore, and none of the top 5 poses by PersScore were native.  Figure 3 shows the average RMSD estimate versus simulation time for all 20 candidate poses.

### 3.6. Conformation Analysis

**Figure 4:**
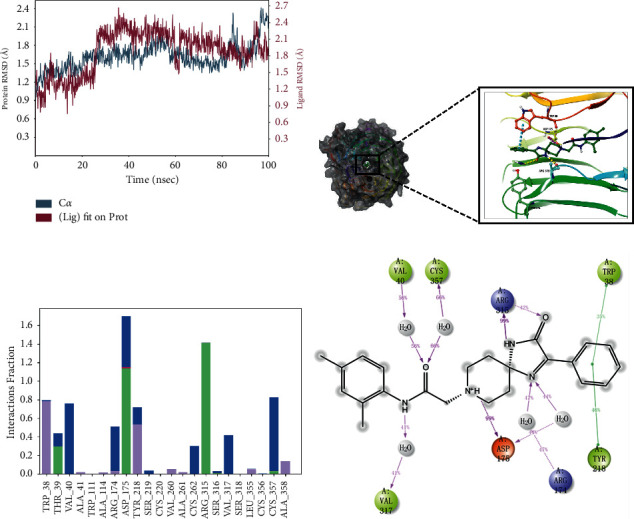
Molecular dynamics simulation and interaction mechanism analysis of G490-0341 with WSB1: (a) root mean square deviation (RMSD) of WSB1 backbone during MD simulation; (b) the 3D plot of the complex; (c) protein-ligand interaction; (d) the 2D plot of the complex.

**Figure 5:**
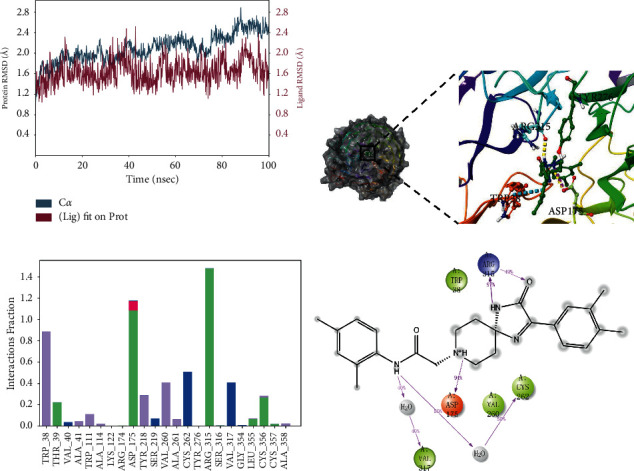
Molecular dynamics simulation and interaction mechanism analysis of G610-0188 with WSB1: (a) root mean square deviation (RMSD) of WSB1 backbone during MD simulation; (b) the 3D plot of the complex; (c) protein-ligand interaction; (d) the 2D plot of the complex.

**Figure 6:**
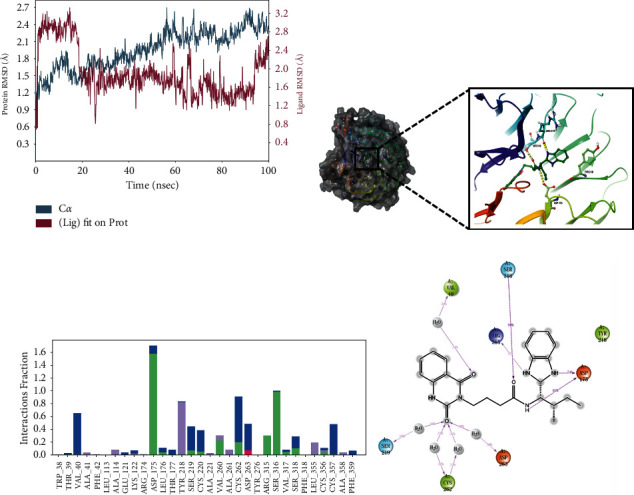
Molecular dynamics simulation and interaction mechanism analysis of Y043-6168 with WSB1: (a) root mean square deviation (RMSD) of WSB1 backbone during MD simulation; (b) the 3D plot of the complex; (c) protein-ligand interaction; (d) the 2D plot of the complex.

**Figure 7:**
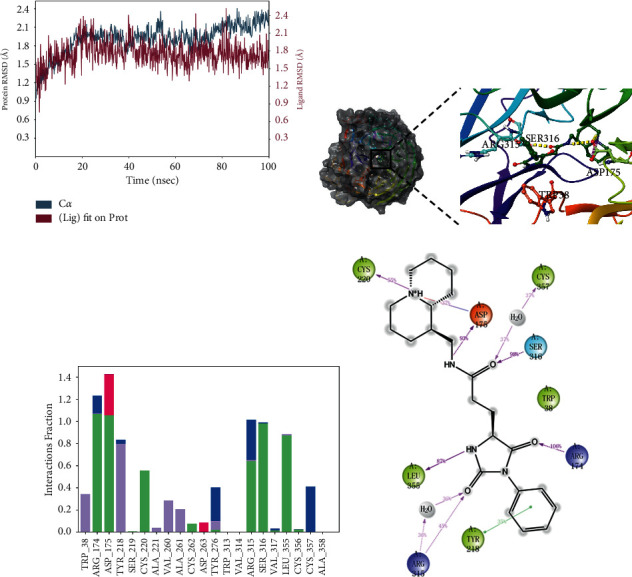
Molecular dynamics simulation and interaction mechanism analysis of Y044-5019 with WSB1: (a) root mean square deviation (RMSD) of WSB1 backbone during MD simulation; (b) the 3D plot of the complex; (c) protein-ligand interaction; (d) the 2D plot of the complex.

To explore the binding modes between the compounds and WSB1 protein, we selected four compounds with more potential in the metadynamics results, including G490-0341, G610-0188, Y043-6168, and Y044-5019, and analyzed them using molecular dynamics (MD) simulations and interaction decomposition. The binding modes of the four complexes are shown in Figures 4–7. Compound G490-0341 formed three hydrogen bonds with residues such as Arg315, Tyr276, and Asp175. An aromatic interaction with residue TRP38 was observed. The reported binding sites for WSB1 might include Arg174, Arg315, and Tyr218. Like G490-0341, G610-0188 (Figure 5), Y043-6168 (Figure 6), and Y044-5019 (Figure 7) also had hydrogen-bonding interactions with residues Asp175 and Arg315. Compound G610-0188, identical to compound G490-0341, had hydrogen bonds with residues Arg315, Tyr276, and Asp175. In addition, both Y043-6168 and compound Y044-5019 formed hydrogen bonds with Ser316 and Y043-6168 formed *π*-*π* interactions with residue Tyr28. Therefore, it was highly likely that the relatively advantageous compound G490-0341 in BPMD forminghydrogen bonds through hydrophobic residues of amino acids such as Arg315, Tyr276, and Asp175 might be more stable and contribute significantly to the formation of the complexes.

## 4. Conclusions

Considering that WSB1 plays an important role in tumor metastasis and tumor cell proliferation, clinically relevant drugs targeting the WSB1 axis as inhibitors are still being investigated. In this study, we predicted the protein structure of WSB1 by Alphafold2 and then used structure-based virtual screening, including AutoDock-GPU and Glide, to select compounds. Through structure-based virtual screening of WSB1 inhibitors, we finally found four compounds with different compound scaffolds such as G490-0341, G610-0188, Y043-6168, and Y044-5019. The binding pose meta-kinetics showed that compound G490-0341 binds tightly to residues Asp175 and Arg315 and is more stable than other compounds. This study will contribute to the further development of WSB1 inhibitors and provide some valuable information for understanding the structure of WSB1 inhibitors. To further study the medicinal properties of these compounds, the ADME/TOX properties of G490-0341 were calculated [50]. The detailed results for the pharmacokinetic parameters and toxicity analyses are shown in Table S1. Computational pharmacokinetics and toxicology studies on G490-0341 suggest that it can be used as a good starting point for further developing and designing new derivatives.

## Figures and Tables

**Table 1:** Docking numerator scores for WSB1 and metadynamics scores.

Entry ID	AutoDock-GPU	Glide SP	Glide XP	PoseScore	ComScore
G610-0009	−10.01	−7.712	−8.674	1.534	−0.766
4464-1021	−10.26	−8.335	−8.645	1.739	−0.441
C776-3318	−10.15	−8.42	−8.643	2.015	−0.53
G796-1782	−10.26	−7.214	−8.462	1.800	−0.925
G610-0205	−10.13	−8.413	−8.456	2.018	−0.257
G610-0036	−10.24	−7.706	−8.333	1.449	−1.921
G490-0341	−10.32	−8.334	−8.31	0.929	−1.676
G610-0191	−10.19	−8.387	−8.256	1.683	−0.922
Y043-6168	−10.15	−7.981	−8.214	1.718	−2.222
G490-0604	−10.37	−8.11	−8.169	1.372	−0.723
G610-0257	−10.88	−8.259	−8.144	1.005	−2.215
G610-0188	−10.71	−8.316	−8.143	0.966	−1.699
G610-0096	−10.16	−8.095	−8.118	3.516	2.516
G610-0149	−10.34	−8.121	−8.073	1.538	−0.682
G610-0007	−10.79	−8.04	−8.057	1.433	−1.827
G490-0586	−10.01	−8.91	−8.021	1.920	−1.3
G610-0156	−10.69	−8.374	−8.009	1.509	−1.596
K784-7081	−12.24	−7.834	−8.008	1.226	−0.354
Y044-5019	−10.02	−8	−8	1.794	−1.651
G490-0627	−10.11	−8.076	−8	1.494	−0.626

## Data Availability

The data used to support the findings of this study are available from the corresponding author upon request.

## Supplementary Materials

Supplementary Materials
*Table S1*. The ADME/TOX prediction of G490-0341.
