# Supplementary material for: Identification of Potential WSB1 Inhibitors by AlphaFold Modeling, Virtual Screening, and Molecular Dynamics Simulation Studies
Source: Evid Based Complement Alternat Med. 2022 May 13;2022:4629392. doi: 10.1155/2022/4629392 (PMC9122669; doi:10.1155/2022/4629392)
Supplement: Supplementary Materials — Table S1. The ADME/TOX prediction of G490-0341. [file 4629392.f1.doc]

**Supporting Information**

**Theoretical evaluation of ADME/TOX properties**

To further study the medicinal properties of these compounds, the ADME/TOX properties of G490-0341 were calculated. The detailed results for the pharmacokinetic parameters and toxicity analyses are shown in Table S1.

G490-0341 expressed drug-like behavior including Molecular Weight less than 500, hydrogen bond donors less than 5, 10 hydrogen bond acceptors, and less than 5 octanol-water partition coefficient(logP). The PSA value of the compound is 73.8, indicating that the compounds have high oral bioavailability. The Caco-2 Permeability is -4.907 and the MDCK Permeability is 5.3e-05. Thus, G490-0341 can be efficiently absorbed in the human intestine, and it can strongly bind to the plasma protein. G490-0341 can slow down the rapid metabolism of drugs by inhibiting CYP2D6 and increasing the time of drug action. However, G490-0341 has some hepatotoxicity. Computational pharmacokinetics and toxicology studies on G490-0341 suggest that it can be used as a good starting point for further developing and designing new derivatives.

| **Property** | **Value** |
| --- | --- |
| Molecular Weight | 390.21 |
| nHA | 6 |
| nHD | 2 |
| logP | 3.031 |
| TPSA | 73.8 |
| Caco-2 Permeability | -4.907 |
| MDCK Permeability | 5.3e-05 |
| CYP2D6 inhibitor | 0.613 |
| Hepatotoxicity | 0.983 |

Table S1. The ADME/TOX prediction of G490-0341
